# Epidermal expression of a sterol biosynthesis gene regulates root growth by a non-cell-autonomous mechanism in *Arabidopsis*

**DOI:** 10.1242/dev.160572

**Published:** 2018-05-15

**Authors:** Eleri Short, Margaret Leighton, Gul Imriz, Dongbin Liu, Naomi Cope-Selby, Flora Hetherington, Andrei Smertenko, Patrick J. Hussey, Jennifer F. Topping, Keith Lindsey

**Affiliations:** Department of Biosciences, Durham University, Durham DH1 3LE, UK

**Keywords:** Epidermal signalling, Sterol biosynthesis, Plant development, *HYDRA1* gene, PIN proteins, Sterol isomerase

## Abstract

The epidermis is hypothesized to play a signalling role during plant development. One class of mutants showing defects in signal transduction and radial patterning are those in sterol biosynthesis. The expectation is that living cells require sterols, but it is not clear that all cell types express sterol biosynthesis genes. The *HYDRA1* (*HYD1*) gene of *Arabidopsis* encodes sterol Δ8-Δ7 isomerase, and although *hyd1* seedlings are defective in radial patterning across several tissues, we show that the *HYD1* gene is expressed most strongly in the root epidermis. Transgenic activation of *HYD1* transcription in the epidermis of *hyd1* null mutants reveals a major role in root patterning and growth. *HYD1* expression in the vascular tissues and root meristem, though not endodermis or pericycle, also leads to some phenotypic rescue. Phenotypic rescue is associated with rescued patterning of the PIN1 and PIN2 auxin efflux carriers. The importance of the epidermis in controlling root growth and development is proposed to be, in part, due to its role as a site for sterol biosynthesis, and auxin is a candidate for the non-cell-autonomous signal.

## Abstract (precis)

**Highlighted Article:** Driving gene expression in different *Arabidopsis* root cell types demonstrates that the importance of the epidermis in regulating root growth is partly due to its role in auxin transport via sterol biosynthesis.

## INTRODUCTION

A key question in plant development is how tissue patterning and cell expansion are coordinated during organ growth. In the root of *Arabidopsis*, for example, radial pattern is highly stereotyped, with predictable numbers of cells in each concentric layer of tissue, with coordination of cell expansion in each layer as the root grows (Dolan et al., 1993). Coordination of cell number and expansion is necessary as plant cells are attached to each other, and failed coordination would likely lead to growth and patterning defects.

The nature of a coordination mechanism remains poorly understood. Mutant screens have identified genes that are essential for correct radial pattern in *Arabidopsis*, providing some insight into the molecular mechanisms involved. For example, the SCARECROW (SCR)/SHORTROOT (SHR) module controls root ground tissue formation, and is characterized by the movement of the SHR protein from the stele, in which the gene is transcribed, to the cortex, where it regulates cell identity (Nakajima et al., 2001). The *keule* and *knolle* mutants exhibit radial defects such as bloated epidermal cells and very short roots, though the genes, which encode interacting components of the membrane trafficking system required for cytokinesis and cell wall construction, are expressed in all cells (Waizenegger et al., 2000; Assaad et al., 2001). Laser ablation experiments also highlight the importance of positional information in the regulation of root tissue patterning through as yet poorly defined signalling mechanisms (van den Berg et al., 1995, 1997). Non-autonomous signalling processes in radial patterning include the movement of transcription factors such as SHR between layers; and brassinosteroid (BR) signalling from the shoot epidermis has been implicated in regulating development of the ground and vascular tissues (Savaldi-Goldstein et al., 2007) and leaf shape (Reinhardt et al., 2007). The CRINKLY4 receptor kinase of maize is expressed in the leaf epidermis and appears to signal to mesophyll cells, probably through an indirect mechanism (Jin et al., 2000; Becraft et al., 2001). In the root, epidermis-derived BR signalling also controls meristem size, through the modulation of, for example, the expression of the MADS-box transcription factor AGL42 in the quiescent centre (Hacham et al., 2011), although the transmitted signal remains unknown.

One class of mutants that exhibit radial patterning defects include the *hydra1*, *fackel/hydra2* and *sterol methyltransferase* (*smt*) mutants, which are defective in sterol biosynthesis (Topping et al., 1997; Jang et al., 2000; Schrick et al., 2002, 2004; Souter et al., 2002). These are distinct from BR mutants, as they cannot be rescued by exogenous BRs (Topping et al., 1997; Schrick et al., 2000). Sterols influence membrane fluidity and permeability, and the activities of membrane-bound proteins (Grandmougin-Ferjani et al., 1997; Hartmann, 1998). Sterols are also implicated in the trafficking and localization of transporter proteins such as the PIN-FORMED (PIN) auxin efflux carriers (Willemsen et al., 2003; Men et al., 2008; Pan et al., 2009; Pullen et al., 2010), and in cell plate construction (Peng et al., 2002; Schrick et al., 2004). Given the water insolubility and presumed lack of mobility of sterols for thermodynamic reasons, the expectation is that they are synthesized in all or the majority of cells, permitting basic cellular functions. However, previous data have suggested that at least some sterol biosynthesis genes, such as *FACKEL*/*HYD2* (Jang et al., 2000; Schrick et al., 2000), are not constitutively expressed. There is also a proposed role for HYD1 in miRNA function, whereby ARGONAUTE1 (AGO1) activity is dependent on HYD1-mediated membrane sterol composition (Brodersen et al., 2012). Mutants such as *hyd1*, *fk*/*hyd2* and *smt1* exhibit significant patterning and growth defects in most cell types, and are typically seedling lethal, suggesting essential roles in all cell types (Jang et al., 2000; Schrick et al., 2002; Souter et al., 2002; Willemsen et al., 2003).

To investigate tissue-dependent sterol function in *Arabidopsis*, we investigated *HYD1* (At1g20050) function using transgenic activation systems to drive expression in different root cell types in the mutant background. This allows the determination of the cell types in which *HYD1* expression is required for correct root development.

## RESULTS AND DISCUSSION

**Fig. 1.**
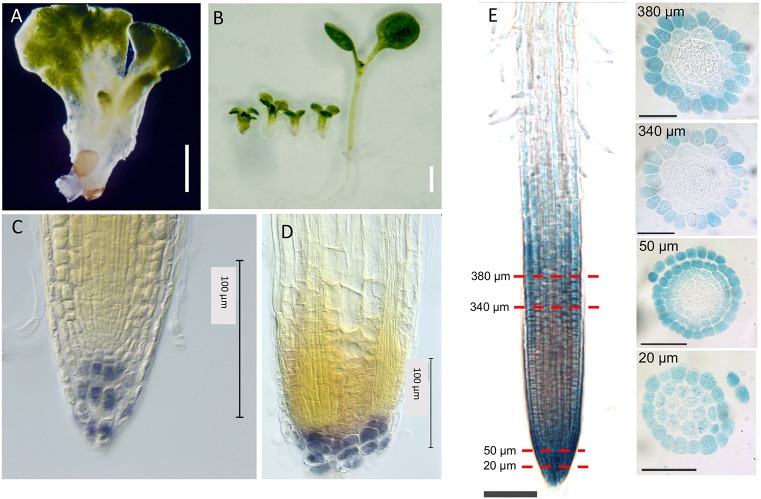
**The *hyd1* mutant and *HYD1* expression.** (A) *hyd1* mutant seedling at 3 dpg. Scale bar: 2 mm. (B) *hyd1* mutant seedlings (left) and wild-type seedling (right) at 5 dpg. Scale bar: 1 cm. (C) Wild-type root stained with lugol at 6 dpg. Scale bar: 100 µm. (D) *hyd1* root stained with lugol at 6 dpg. Scale bar: 100 µm. (E) *proHYD1::GUS* expression in wild-type seedling root at 7 dpg. Red lines indicate the section distance from the root apex. Scale bars: 75 µm.

*hyd1* seedlings exhibit abnormal morphogenesis and cell patterning and growth in the root (Fig. 1; Topping et al., 1997; Souter et al., 2002), with defective radial pattern seen during embryogenesis (Topping et al., 1997; Souter et al., 2002, 2004). Mutant seedlings typically develop multiple cotyledons of aberrant shape, a short hypocotyl and very short root (Fig. 1A,B). The root has a defective apical meristem associated with aberrant patterning of surrounding cells (epidermis, columella, ground tissue, vascular tissue; Fig. 1C,D; Fig. S1). Given the defects across several cell types, and the expectation that all cells contain sterols, we monitored spatial activity of the expression of a 2 kb fragment of the *HYD1* gene promoter as a transcriptional fusion reporter with a β-glucuronidase (GUS, *uidA*) gene in transgenics. We have previously demonstrated that this 2 kb *HYD1* gene promoter fragment driving the *HYD1* cDNA is sufficient to complement the *hyd1* mutant (Souter et al., 2002; Fig. S2), and is expected to contain the major regulatory elements necessary for regulating *HYD1* transcription. The expression pattern in the primary root of transgenics is shown in Fig. 1E. Unexpectedly, results show a non-homogeneous expression pattern, localized principally to the lateral root cap, epidermis of root elongation zone and, less strongly, in the root differentiation zone (especially in trichoblast files), with some detectable expression in the root cortex. This is broadly consistent with cell expression profiling visualized in the Toronto expression profiling browser tool (bar.utoronto.ca/eplant/; Winter et al., 2007) based on data from Brady et al. (2007; Fig. S3), which shows strongest expression in the epidermal cells of the meristematic zone, with lower expression in the epidermis of the maturation zone, cortex and provascular tissues, and little detectable expression in mature vascular cells. There is also a reduced expression in the epidermis and cortex at the transition between meristematic and elongation zones. These observations raise the question of how such an expression pattern leads to radial patterning defects across a wider range of tissues in the primary root of the *hyd1* mutant.

Although sterols are transported between intracellular membrane compartments via lipid transfer proteins (Saravanan et al., 2009), there is no evidence that they are transported between cells. The question then is how can localized expression of the *HYD1* gene, which is essential for radial patterning throughout embryogenesis and post-embryonic growth, mediate the development of cell types in which it is not active? We have shown, for example, that vascular patterning is abnormal, and PIN1 localization is aberrant in the vascular cells, even though *HYD1* is not expressed in mature vascular cells, and only weakly expressed in xylem pole pericycle and possibly phloem companion cells (Fig. S3; Pullen et al., 2010). This suggests that a non-cell-autonomous signal is transferred from *HYD1*-expressing cells to non-expressing cells, to mediate wild-type tissue patterning.

To understand which cells might be sufficient and/or necessary for *HYD1* expression to mediate processes essential for root growth and development, the full-length *HYD1*-coding sequence (Topping et al., 1997) was cloned behind a variety of promoters and the UAS for use in a *mGAL4-VP16-GFP* enhancer trap transactivation system (Laplaze et al., 2005). This can be used to drive *HYD1* transcription in different root cell types: the columella and QC, the epidermis, the endodermis, the pericycle, and vascular cells. This enhancer trap activation system relies on the cell type-specific expression of the GAL4 transcription factor-activated UAS, which is regulated by local enhancers in the genome that activate *HYD1* transcription. The vector also includes a GAL4-responsive *GFP* gene to visualize expression patterns and confirm spatial specificity (Laplaze et al., 2005). *Promoter::HYD1* and *UAS::HYD1* fusions were transformed into wild-type *Arabidopsis* and crossed with the *hyd1* heterozygotes, and homozygous mutant seedlings containing the *promoter/UAS::HYD1* fusions were identified by genotyping and microscopy analysis. Several independent transgenics were generated and typical expression patterns were identified in specific lines.

**Fig. 2.**
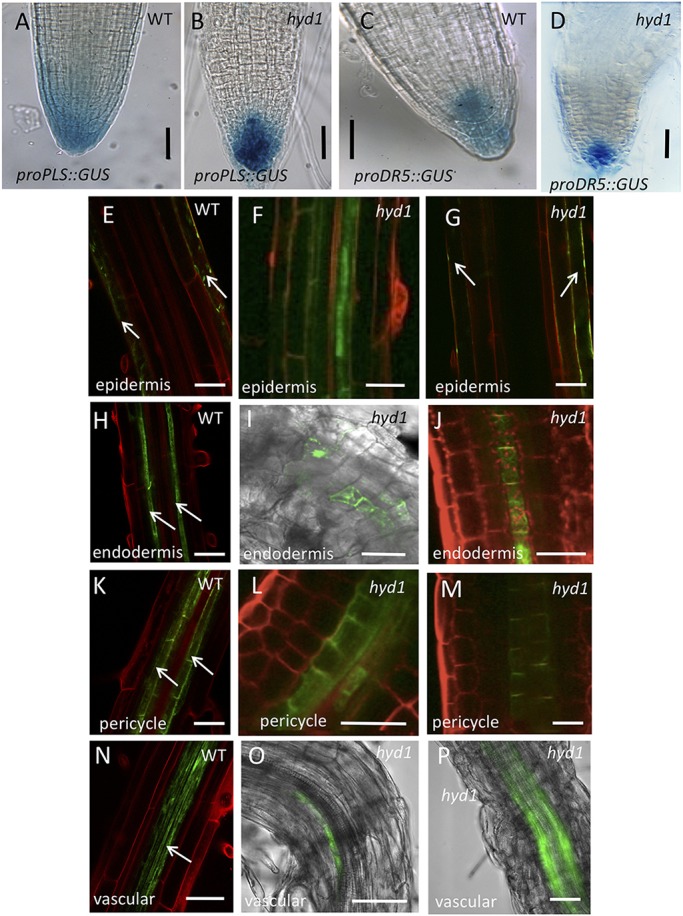
**Cell type expression of promoters in wild type and *hyd1* mutants.** (A,B) *proPLS::GUS* expression in roots of wild-type (A) and *hyd1* (B) seedlings at 7 dpg. (C,D) *DR5::GUS* expression in roots of wild-type (C) and *hyd1* (D) seedlings at 7 dpg. (E-G) GFP expression in epidermal cells in the GAL4 driver line J2551 in wild-type (E) and *hyd1* (F,G) roots at 7 dpg. In F and G, the surface of the root is imaged, showing variability in GFP expression. (H-J) GFP expression in endodermal cells in the GAL4 driver line J3611 in wild-type (H) and *hyd1* (I,J) seedling roots at 7 dpg. (K-M) GFP expression in pericycle cells in the GAL4 driver line J0272 in wild-type (K) and *hyd1* (L,M) seedling roots at 7 dpg. (N-P) GFP expression in vascular cells in the GAL4 driver line J0661 in wild-type (N) and *hyd1* (O,P) seedling roots at 7 dpg. Arrows indicate expression in epidermis (E,G), endodermis (H), pericycle (K) and vascular tissues (N). Scale bars: 50 µm.

For expression in the columella and QC, we used the *POLARIS* (*PLS*) promoter (Casson et al., 2002) and the synthetic promoter *DR5* (Sabatini et al., 1999). The respective promoter-GUS expression patterns in both wild-type and *hyd1* mutant root tips are shown in Fig. 2A-D. For epidermal and lateral root cap expression, we used the GAL4 driver line J2551 (Fig. 2E-G); for endodermis, line J3611 (Fig. 2H-J); for pericycle, line J0272 (Fig. 2K-M); and for vascular cells, line J0661 (Fig. 2N-P). Expression in the *hyd1* mutant reflects the aberrant tissue patterning, but both *promoter::GUS* and *UAS::HYD1:GFP* lines were identified that exhibited expression in the expected cell types. Examples are given in Fig. 2 of different seedling lines exhibiting the various *UAS::HYD1:GFP* expression patterns. These results provide the basis for the use of the promoter/UAS regulatory sequences to drive *HYD1* transcription in specific cell types, to determine the effects on root development.

**Fig. 3.**
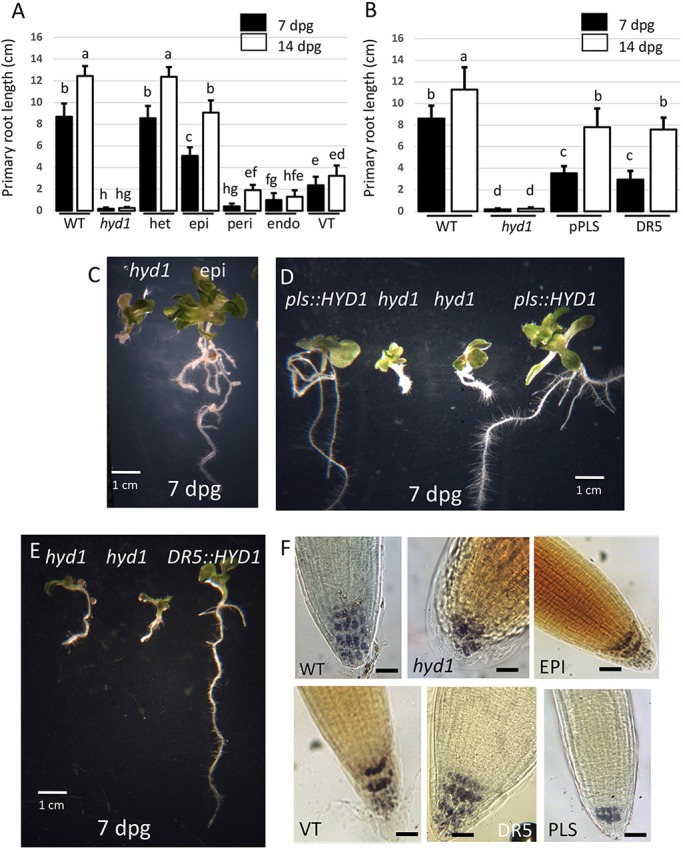
**Effect of cell type-specific expression of the *HYD1* gene on root growth and columella organization.** (A,B) Primary root length of wild-type (WT), *hyd1*/WT heterozygous (het), *hyd1* homozygous (*hyd1*) and transgenic *hyd1* seedlings expressing *HYD1* in the epidermis (epi), pericycle (peri), endodermis (endo), vascular tissues (VT) and root tips under the control of the *PLS* and *DR5* promoters at 7 and 14 dpg. (C-E) *hyd1* mutant and transgenic *hyd1* seedlings expressing *HYD1* in the GAL4 driver line J2551 in epidermal cells (epi; C) and in root tips (*proPLS::HYD1*, D; *DR5::HYD1*, E) at 7 dpg. Scale bars: 1 cm. (F) Root tips of wild-type (WT), *hyd1* and transgenic *hyd1* seedlings expressing *HYD1* in the GAL4 driver line J2551 in epidermis (EPI), in line J0661 in vascular tissues (VT), and in root tips under the control of the *DR5* and *PLS* promoters at 7 dpg. Scale bars: 50 µm. Data are mean±s.d. Common letters above bars indicate no significant difference (*P*>0.05; Tukey pairwise comparisons, *n*=9).

Genetically homozygous mutant seedlings expressing the *HYD1*-coding region in different cell types were grown for up to 21 days on vertical agar plates for phenotypic and growth analysis. Although the mean length of wild-type roots was ∼12.4 cm at 14 dpg, and for the *hyd1* homozygous mutants was typically approximately less than 0.5 cm at 14 dpg, there were significant differences in the effects of expressing *HYD1* in different cell types (Fig. 3A,B). The most significant restoration of primary root growth was in *hyd1* seedlings expressing the *HYD1* gene in the epidermis (i.e. J2551≫HYD1; mean primary root length 9.1 cm at day 14), in the vascular tissues (i.e. J0661≫HYD1; mean primary root length 3.2 cm at day 14), and in the root columella and lateral root cap (DR5::HYD1, PLS::HYD1; mean primary root length up to ca. 7.8 cm at day 14). In each of these lines, primary root length was restored to ∼60-80% that of wild type by 14 dpg for epidermal expression, and with only ∼30% wild-type growth seen with *HYD1* expression in vascular tissue (Fig. 3A,C). Fifty-five to 65% of wild-type root growth was seen in seedlings with expression in the root columella and lateral root cap (Fig. 3B,D,E).

Associated with significant restoration of root morphology and growth is an improved patterning of cells in the root tip, seen as a regularized organization of the starch-containing columella, though still with variable tiers of cells (Fig. 3F). Wild-type seedlings most commonly had four tiers of columella cells [3.8±0.4 (mean±s.d.), *n*=10], with cells of regular shape. In the *hyd1* mutant, columella patterning is aberrant, with reduced layers (1.5±0.7, *n*=10) and disorganized cell shape. Although growth rescue was best when *HYD1* was expressed in the epidermis and root tip under either the *PLS* or *DR5* promoters (Fig. 3A,B), *HYD1* expression from the *PLS* promoter was less successful in rescuing columella pattern (Fig. 3F; 2.7±0.6, *n*=10). Vascular tissue expression produced similar results (2.8±0.4, *n*=10), but better patterning and cell shape seen when expressed in the epidermis (3.4±0.5, *n*=10) or under the *DR5* promoter (3.8±0.4, *n*=10).

These results show that the epidermal expression of *HYD1* plays a major role, but alone is insufficient for correct root development; and expression in other cells contributes to growth and patterning. Given the partial but significant growth rescue by *HYD1* expression in vascular tissues (to ∼30% of wild type, Fig. 3A), but still with poor columella rescue, there exists the possibility that sterol synthesis in different cell types may contribute to different components of root development (e.g. growth versus cell patterning in the distal part of the root). There is only very limited rescue of aerial parts observed when *HYD1* transcription is driven in root tissues, though *DR5::HYD1* (which is expressed in aerial parts) does lead to some rescue of leaf size and shape (Fig. S4).

Root growth and cell patterning, including columella organization, have been linked to auxin concentration and response in the root (Sabatini et al., 1999; Aida et al., 2004). Sterols are required for correct auxin-mediated gene expression (Souter et al., 2002, 2004) and for PIN localization, including in the *hyd1* and *smt* mutants (Carland et al., 2010; Pullen et al., 2010). PIN proteins act as auxin efflux carriers, allowing directional auxin transport to establish gradients across tissues, often with developmental or tropic consequences. Correct membrane sterol composition has been shown to be required for correct PIN2 polarity and gravitropic response (Men et al., 2008). To determine whether the activation of the *HYD1* gene in specific cell types is associated with a restoration of PIN localization, PIN1 and PIN2 were immunolocalized in the mutant, wild-type and transgenic lines expressing *HYD1* under control of cell type-specific promoters.

**Fig. 4.**
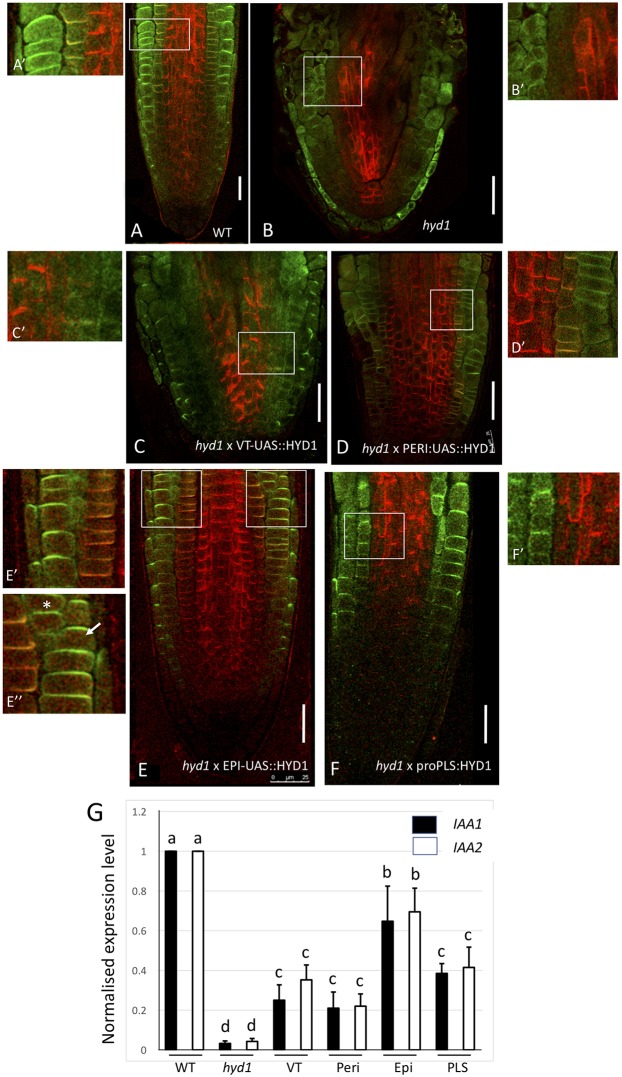
**Effect of cell type-specific expression of the *HYD1* gene on PIN proteins and auxin gene expression.** PIN1 (red) and PIN2 (green) immunolocalization in wild-type root (A,A′), in *hyd1* mutant root (B,B′), in *hyd1* mutant root expressing *HYD1* in vascular tissues (VT) (C,C′), in *hyd1* mutant root expressing *HYD1* in pericycle cells (D,D′), in *hyd1* mutant root expressing *HYD1* in epidermal cells (E,E′,E″; with E′ from left box in E and E″ from right box in E) and in *hyd1* mutant root expressing *HYD1* in root tips (*PLS* gene promoter; F,F′) at 7 dpg. Scale bars: 50 µm. Arrow in E″ indicates PIN2 in epidermis, asterisk indicates PIN2 in cortex. (G) Normalized expression of *IAA1* (black bars) and *IAA2* (open bars) in whole seedlings of *hyd1* mutant (HYD1), in roots of wild-type seedlings (WT), in roots of seedlings expressing *HYD1* in vascular tissues (VT), in roots of seedlings expressing *HYD1* in pericycle cells (Peri), in roots of seedlings expressing *HYD1* in epidermal cells (Epi) and in roots of seedlings expressing *HYD1* in root tip (PLS) relative to wild type (value 1), determined by qRT-PCR. Data are mean±s.d. of four biological replicates. Common letters above bars indicate no significant difference (*P*>0.05; Tukey pairwise comparisons).

Results presented in Fig. 4 show that, as expected, the wild-type root tip shows PIN1 localized to the basal region of cells in the stele of the root, and PIN2 localized to the apical side of epidermal cells (Fig. 4A). In the *hyd1* mutant, both abnormal cellular patterning and loss of polar PIN1 and PIN2 localization are evident (Fig. 4B). Cellular patterning in seedlings expressing *HYD1* in the vascular tissues (Fig. 4C) or pericycle (Fig. 4D) is poorly restored, and PIN expression and localization is variable and associated with relatively poor primary root growth (Fig. 3A). In seedlings expressing *HYD1* in the epidermis, cellular patterning is similar to wild type, as is the localization of PIN2 and PIN1 (Fig. 4E). This is associated with relatively long primary roots in these seedlings (Fig. 3A). In *proPLS::HYD1* seedlings (Fig. 4F), radial patterning of the root is restored close to wild type, with an improvement of PIN localization compared with either the *hyd1* mutant or, for example, the vascular tissue line. The auxin-regulated genes *IAA1* and *IAA2* are known to be poorly expressed in the *hyd* mutants (Souter et al., 2004; Fig. S5) but activation of *HYD1* in the epidermis, and also to some extent in the vascular tissues and root tip (*PLS::HYD1*) leads to some recovery of the expression levels of both genes (Fig. 4G).

Our results suggest that the epidermis plays an important role in controlling growth as a site for sterol biosynthesis, and this involves a non-autonomous signalling pathway, for which auxin is a strong candidate. Previous evidence demonstrated a non-autonomous role for the epidermis in BR synthesis (Hacham et al., 2011), but the signal involved was not identified. Our data suggest that at least one coordinating signal across tissues is auxin, and the role of sterols in this context is to mediate correct localization and function of the PIN proteins, which are responsible for directional auxin transport. We cannot exclude the possibility that low *IAA* gene expression in the *hyd1* mutant is due to reduced auxin biosynthesis, but previous work has shown that the mutants are capable of activating auxin accumulation (either by modulation of biosynthesis or transport mechanisms) by blocking ethylene signalling (Souter et al., 2004; Pullen et al., 2010). Given the established link between auxin transport and sterols, we believe the simplest model for our observations involves effects on auxin transport rather than biosynthesis. Sterols are known to control PIN polarization, and we suggest that sterols are likely functioning in a non-autonomous fashion by mediating auxin gradient establishment, which in turn controls patterning and growth, through, for example, activation of the PLT/WOX5 mechanism (Aida et al., 2004; Sarkar et al., 2007). Exogenous auxin does not rescue the *hyd* mutant phenotype, supporting the view that gradients of auxin rather than absolute levels are required for correct development; and the *hyd1* mutant lacks PIN3 proteins (Souter et al., 2002), which in wild type accumulate distal to the quiescent centre and distribute auxin both down into the columella and laterally towards the epidermis and cortex (Friml et al., 2002). *hyd1* also shows defective PIN1 and PIN2 localization and auxin patterning, associated with defective cell patterning (Pullen et al., 2010). The epidermis appears crucial as a site of sterol biosynthesis via HYD1 – the *hyd1* mutants fail to accumulate key sterols (Souter et al., 2002), and expression of the *HYD1* gene specifically in the epidermis significantly rescues root growth and patterning of cells in the root tip.

Interestingly, *HYD1* expression in the root cap (both columella and lateral root cap cells) also leads to significant root growth rescue, presumably by promoting PIN activity there to ensure stem cell niche activity - columella patterning is rescued, as well as root growth. Expression in the pericycle, endodermis or vascular tissues, on the other hand, has limited effects on root growth (Fig. 3), pointing to a mechanism distinct to, for example, the role of gibberellins in the endodermis (Ubeda-Tomás et al., 2008, 2009). These results show that the importance of the epidermis in regulating root growth can at least in part be explained by its role in auxin transport via sterol biosynthesis.

## MATERIALS AND METHODS

### Plant material

The *hyd1* mutant was identified in an insertional mutagenesis screen as described previously (Topping et al., 1997; Souter et al., 2002). The full-length *HYD1* cDNA sequence was cloned into the vector pCIRCE, and fused to the promoters *DR5* (Sabatini et al., 1999), *PLS* (Casson et al., 2002) or *UAS* (Laplaze et al., 2005). The constructs were introduced into *Arabidopsis thaliana* by floral dip transformation (Clough and Bent, 1998). Homozygous T2 lines containing the *UAS:HYD1* were crossed with the GAL4 driver lines J2551 (epidermal and lateral root cap expression), J3611 (endodermis), J0272 (pericycle) and J0661 (vascular cells), kindly provided by Dr Jim Haseloff (Cambridge University, UK). Information on the cell specificity of expression of the enhancer trap drivers is available on the Haseloff website (www.plantsci.cam.ac.uk/Haseloff). Plants homozygous for all *HYD1* constructs were crossed respectively with plants heterozygous for the *hyd1* mutation, and selfed to identify progeny that was homozygous for both the original *hyd1* mutation and the *HYD1* fusion transgene for further analysis. For growth assays, seeds were stratified, surface sterilized and grown on vertical agar plates containing half-strength Murashige and Skoog medium, as described previously (Topping et al., 1997).

### Gene expression analysis

RNA was extracted from seedling tissues (either whole seedlings or roots, according to the specific experiment), and gene expression measured by quantitative RT-PCR, with *ACTIN3* as an internal standard, as described previously (Rowe et al., 2016).

### Immunofluorescence microscopy and imaging

*Arabidopsis* roots were fixed for 60 min at room temperature with 4% (w/v) paraformaldehyde in 0.1 M PIPES (pH 6.8), 5 mM EGTA, 2 mM MgCl_2_ and 0.4% Triton X-100. The fixative was washed away with PBST buffer, and cells were treated for 8 min at room temperature with the solution of 2% (w/v) Driselase (Sigma) in 0.4 M mannitol, 5 mM EGTA, 15 mM MES (pH 5.0), 1 mM PMSF, 10 μg ml^−1^ leupeptin and 10 μg ml^−1^ pepstatin A. Thereafter, roots were washed twice for 10 min each in PBST then in 1% (w/v) BSA in PBST for 30 min and incubated overnight with a primary antibody. The primary antibodies were rabbit anti-PIN1 and guinea pig anti-PIN2 (both used at 1:150, provided by Prof. Klaus Palme, University of Freiburg, Germany). Specimens were then washed three times for 90 min in PBST and incubated overnight with goat anti-mouse TRITC and anti-rabbit FITC-conjugated secondary antibodies diluted 1:200. After washing in the PBST buffer, specimens were mounted in the Vectashield (Vector Laboratories) mounting medium. Images were acquired using Leica SP5 laser confocal scanning microscope using excitation at 488 nm line of Argon laser for FITC or 561 nm excitation of solid-state laser for TRITC. The emitted light was collected at 505-550 nm or 570-620 nm, respectively.

Light micrographs were acquired using a Zeiss Axioskop microscope (Carl Zeiss) equipped with Photometrics CoolSNAP cf colour digital camera (Roper Scientific) and OpenLab3.1.1 software (Improvision). GFP signal in roots was imaged using a Leica SP5 confocal microscope using 488 nm line of argon laser and emission was collected between 505 and 530 nm. The roots were mounted in double-distilled water under a large (25×50 mm) zero-thickness coverslip. Lugol staining was carried out as described previously (Souter et al., 2002).

### Statistical analysis

All statistical analyses were performed in IBM SPSS Statistics for Windows, Version 22. *P*<0.05 was considered significant. The one-way analysis of variance (ANOVA) and Tukey pairwise comparison post-hoc tests were used to determine significance between the means of three or more independent groups.

## Supplementary Material

Supplementary information
