# Supplementary material for: Epidermal expression of a sterol biosynthesis gene regulates root growth by a non-cell-autonomous mechanism in Arabidopsis
Source: Development. 2018 May 15;145(10):dev160572. doi: 10.1242/dev.160572 (PMC6001376; doi:10.1242/dev.160572)
Supplement: Supplementary information [file develop-145-160572-s1.pdf]

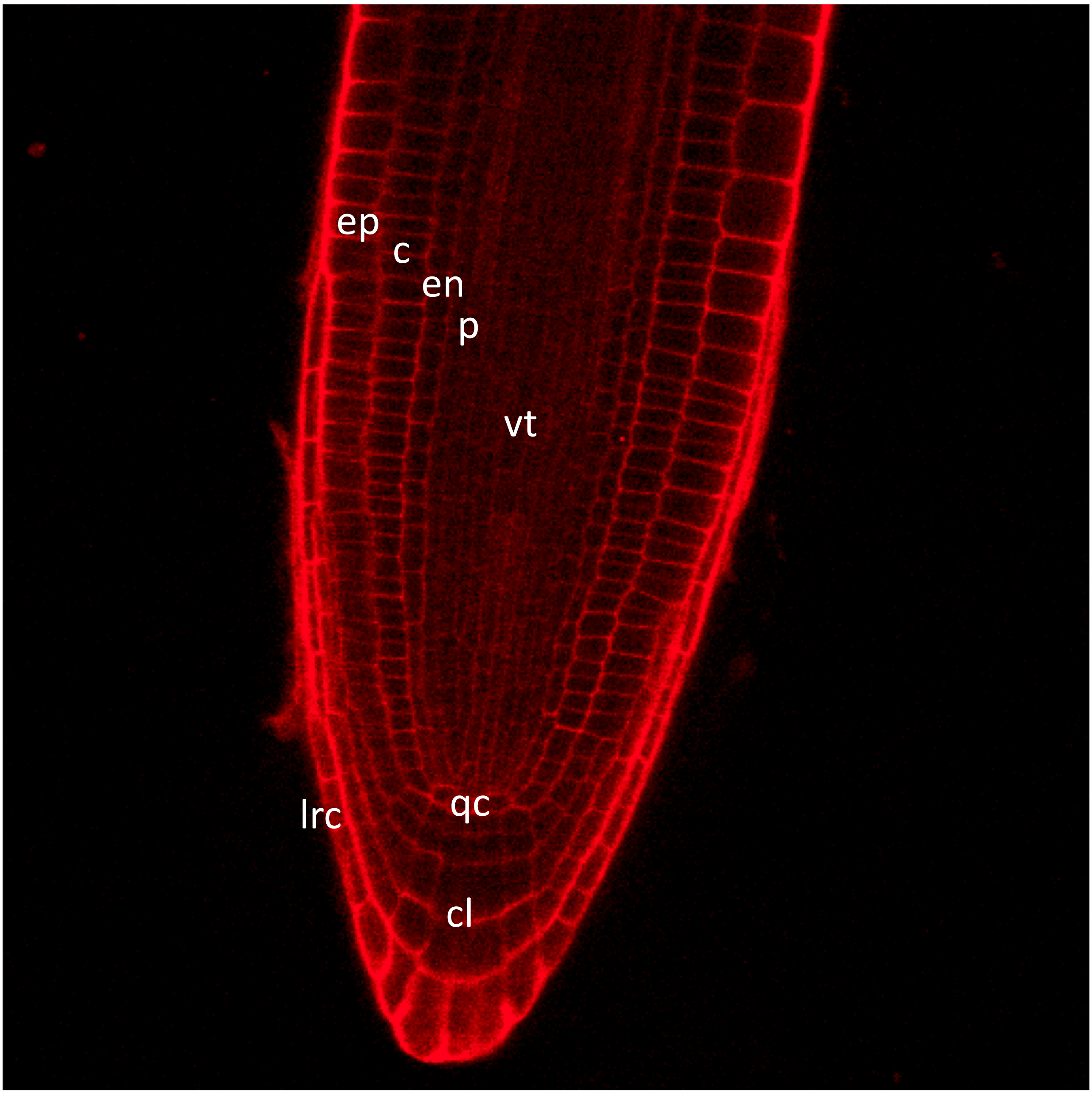

Supplementary Figure 1. Cell types in the Arabidopsis root tip.
ep = epidermis
c = cortex
en = endodermis
p = pericycle
vt = vascular tissues
qc = quiescent centre
lrc = lateral root cap
cl = columella

Development • Supplementary information

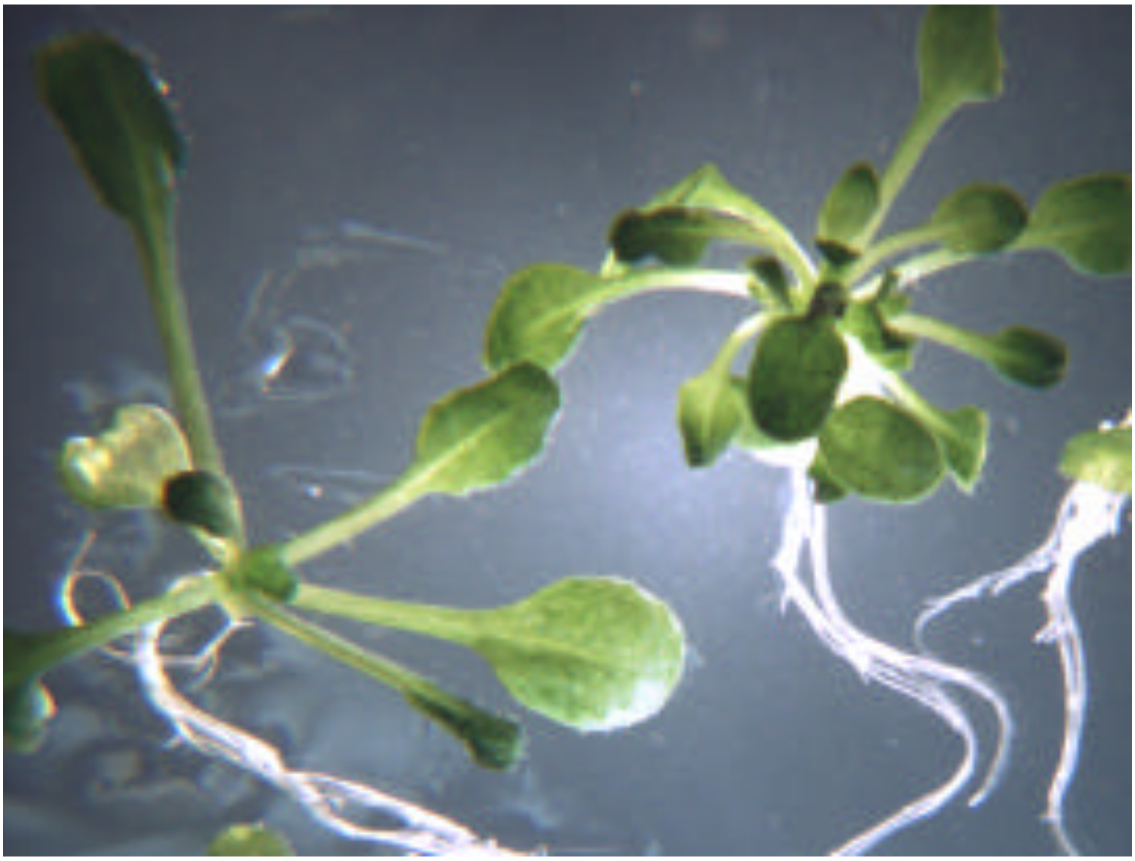

Supplementary Figure 2. *hyd1* mutant seedlings rescued by transgenic expression of proHYD1::HYD1, showing more normal growth of shoots and roots.

For further information see Souter *et al*. (2002)

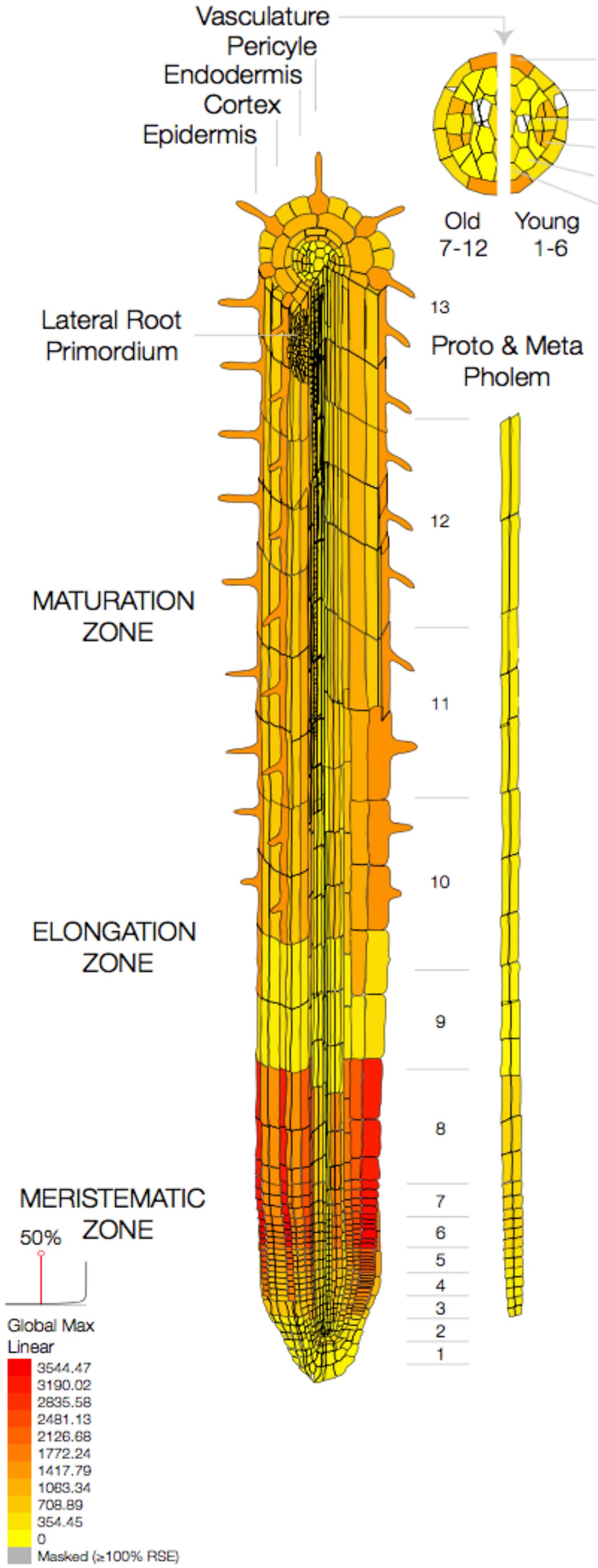

Supplementary Figure 3. *HYDRA1* gene expression visualised in the Toronto expression profiling browser tool (http://bar.utoronto.ca/eplant/; Winter et al., 2007) based on data from Brady et al. (2007).

Development • Supplementary information

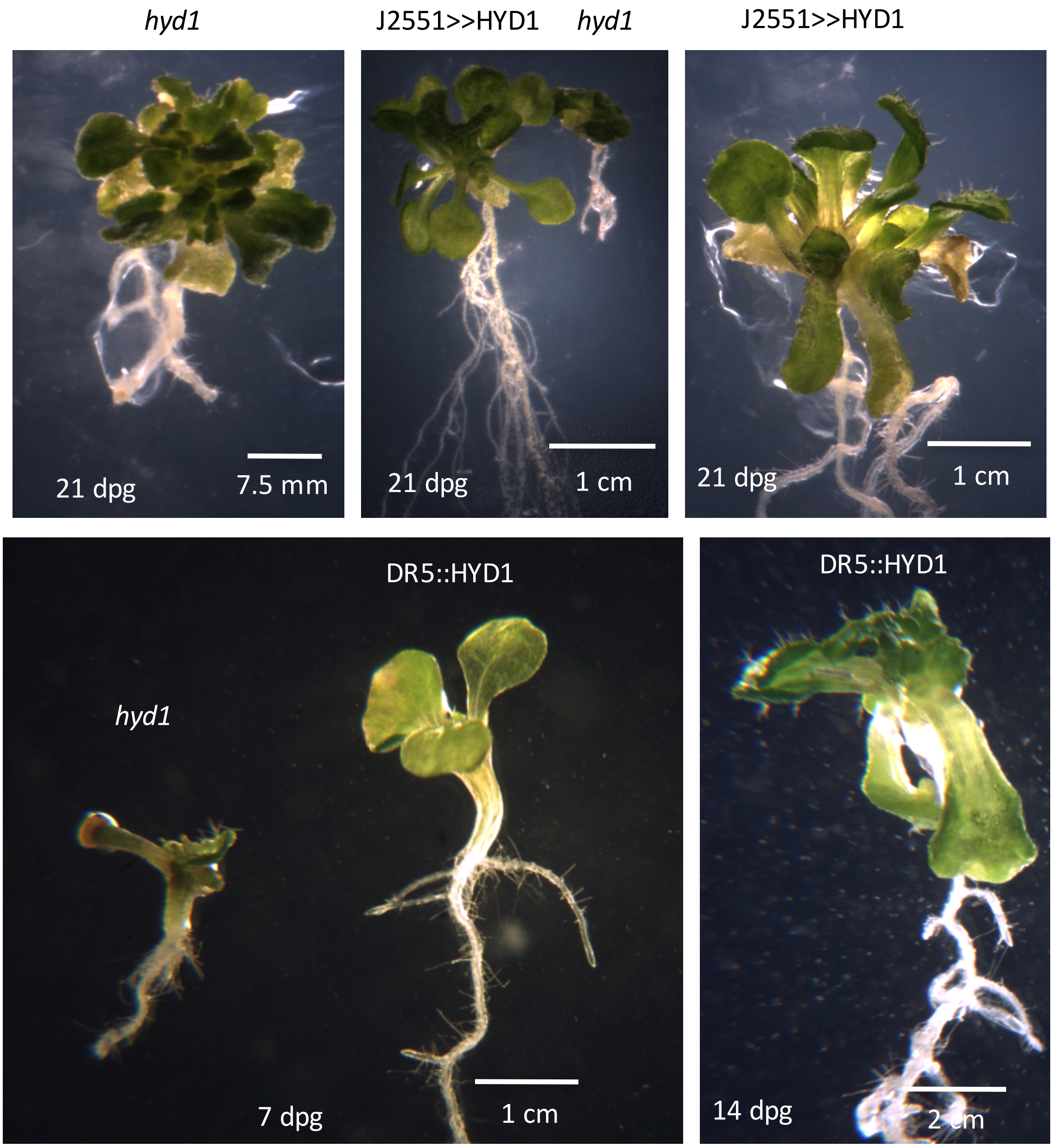

Supplementary Figure 4. Examples of seedlings showing limited rescue of aerial parts.
Upper panels: *hyd1* mutant seedlings, and seedlings with the *HYD1* cDNA driven in epidermal tissues (J2551>>HYD1), showing poor rescue of aerial parts. Seedlings at 7 dpg.
Lower panels: *hyd1* mutant seedlings, and seedlings with the *HYD1* cDNA driven by the DR5 promoter DR5::HYD1) showing limited rescue of aerial parts. Seedlings at 7 (left) and 14 (right) dpg.

Development • Supplementary information

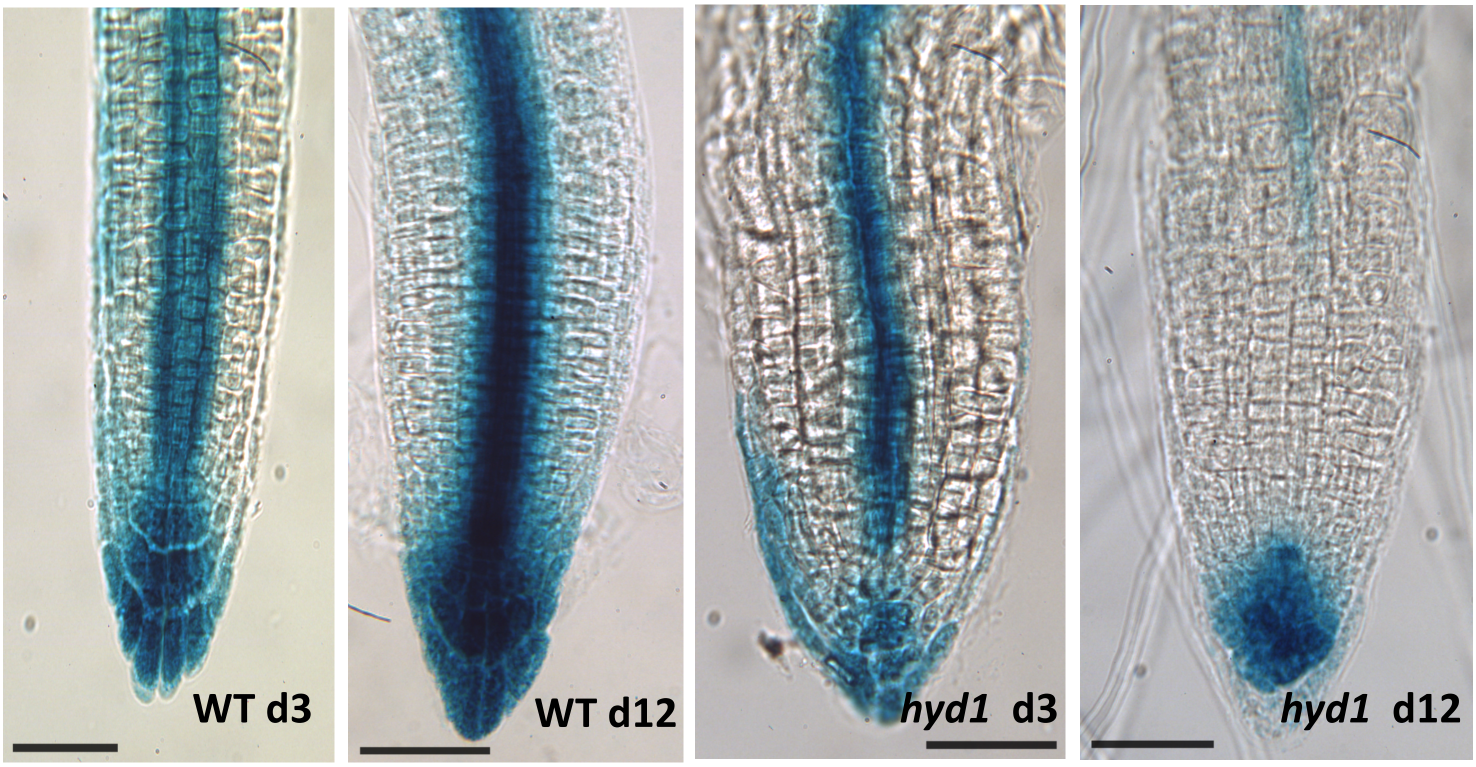

Supplementary Figure 5. *IAA2::GUS* expression in wildtype (WT) and *hyd1* seedling root tips, at either d3 or d12 post germination. Bar = 50 µM
